# Functional immune profiling reveals CD4 ^+^ T cell dysregulation in coeliac disease

**DOI:** 10.1111/imcb.70132

**Published:** 2026-05-17

**Authors:** Anthony J Farchione, HoChan Cheon, David Vremec, Julika Neumann, Gwenny M Verstappen, Melinda Y Hardy, Mai B Margetts, Lauren J Howson, Lee M Henneken, Maureen Forde, Jason A Tye‐Din, Susanne Heinzel, Philip D Hodgkin, Vanessa L Bryant

**Affiliations:** ^1^ Walter and Eliza Hall Institute of Medical Research Parkville VIC Australia; ^2^ Department of Medical Biology University of Melbourne Parkville VIC Australia; ^3^ Department of Clinical Immunology & Allergy The Royal Melbourne Hospital Parkville VIC Australia; ^4^ Department of Gastroenterology The Royal Melbourne Hospital Parkville VIC Australia

**Keywords:** CD4^+^ T cells, Coeliac disease, IL‐2, Immune dysregulation, Mathematical modelling, T cell activation

## Abstract

T cells integrate signals from antigen and costimulatory receptors to calibrate response magnitude and quality, with genetically encoded programs shaping activation thresholds for immune tolerance and feedback regulation. Coeliac disease (CeD) is an autoimmune disorder with well‐defined genetic risk and immune dysregulation triggered by dietary gluten. However, how genetic risk translates into cell‐intrinsic functional variation, particularly within the naïve T‐cell compartment, remains poorly defined. Here, we developed the *T cell momentum assay*, a quantitative functional profiling platform combining standardized T‐cell activation with defined stimulus withdrawal to measure proliferation, survival and activation dynamics over time. Integrated with the Cyton2 mathematical model, this approach infers cellular fate programs from population‐level dynamics, enabling high‐resolution analysis of intrinsic T‐cell behavior. Applying this assay to naïve T cells from individuals with CeD and healthy donors (HDs), we identified disease‐associated abnormalities predominantly in CD4^+^ T cells, including hypoproliferation, reduced IL‐2 secretion, impaired survival and delayed downregulation of CD69, indicating prolonged activation and impaired feedback regulation. Distinct early alterations in CD8^+^ T cells were also observed. These abnormalities were present in both newly diagnosed individuals and those on a gluten‐free diet, supporting a cell‐intrinsic phenotype not solely attributable to active inflammation and is consistent with altered baseline immune function. Together, our findings reveal previously unrecognized alterations in naïve T‐cell programming in CeD, linking inherited immune variation to functional dysregulation beyond antigen‐specific responses. More broadly, the momentum assay offers a scalable, model‐informed framework to detect subtle early T cell dysregulation and functionally stratify immune variation across autoimmune diseases.

## Graphical abstract

The T cell momentum assay quantifies division, survival and activation decay to reveal intrinsic CD4^+^ programming defects marked by prolonged activation and impaired feedback control in coeliac disease. This scalable platform provides a functional screen for early T cell dysregulation across autoimmune and immune‐mediated diseases.

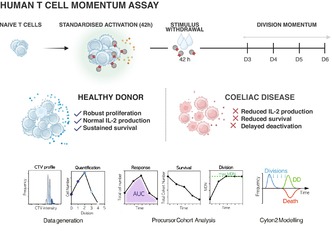

## INTRODUCTION

Naïve T cells are the cornerstone of adaptive immunity, comprising antigen‐inexperienced lymphocytes poised to respond to novel challenges. Because they have not undergone antigen‐driven clonal expansion or effector commitment, these cells retain molecular and functional features that reflect inherited immune programming, providing a window into how germline genetic variation shapes baseline immune setpoints. As such, naïve T cells offer a unique opportunity to reveal cell‐intrinsic immune dysregulation underlying complex diseases, where risk is polygenic and the functional consequences of inherited variation remain poorly defined.

Coeliac disease (CeD) is a chronic immune‐mediated enteropathy triggered by exposure to dietary gluten in genetically susceptible individuals.^1^ It is characterized by an acquired adaptive immune response to post‐translationally modified (deamidated) gluten peptides^2^ presented by HLA‐DQ2.5, 2.2 and/or HLA‐DQ8 molecules to CD4^+^ T cells.^3^ Once established, this response drives rapid release of interleukin‐2 (IL‐2) and acute gastrointestinal symptoms within 2 h of gluten exposure, followed by specific expansion of gluten‐reactive CD4^+^ T cells^4^ and a broader, non‐specific activation of gut‐homing CD8^+^ T cells within 1 week.^5^ The gluten‐specific CD4^+^ T cell pool persists as long‐lived effector memory clonotypes in both the gut and circulation, reactivating upon re‐exposure to gluten.^6^ CD8^+^ intraepithelial lymphocytes also contribute to tissue pathology, acquiring cytolytic NK‐like features in proinflammatory environments and are implicated in mediating the enteropathy of CeD.^2^ In addition to gluten‐specific responses, autoreactive CD4^+^ T cells directed against non‐gluten antigens have also been implicated, suggesting broader defects in T‐cell tolerance and regulation.^7^, ^8^ Sustained gluten exposure results in the characteristic histological features of CeD in the small intestine, including diagnostic villous atrophy and crypt hyperplasia.^9^

Genetic studies have established that CeD risk is strongly influenced by HLA‐DQ2.5 and HLA‐DQ8 alleles,^10^ with over 40 additional non‐HLA loci contributing to disease susceptibility.^11^, ^12^, ^13^, ^14^ Many of these loci are enriched in pathways regulating T‐cell activation, costimulation and cytokine signaling, including *IL2, PTPN2, CD28* and *STAT4*, pointing to broader dysfunction in T‐cell responses. Additionally, transcriptomic analyses of gluten‐specific CD4^+^ T cells from individuals with CeD further support widespread dysregulation of activation‐associated pathways, suggesting that intrinsic abnormalities in T‐cell function may contribute to disease risk and progression.^15^ Given this strong polygenic architecture, particularly within pathways regulating T cell activation and IL‐2 signaling, we hypothesized that naïve T cell behavior reflects genetically encoded functional setpoints.

To date, mechanistic studies in CeD have primarily focused on antigen‐experienced, gluten‐reactive CD4^+^ T cells either following gluten challenge or stimulated ex vivo. While informative, these approaches do not address whether polyclonal naïve T cells, prior to antigen exposure, exhibit intrinsic functional differences. Given the polygenic nature of CeD, we hypothesized that subtle alterations in baseline T cell programming may shape immune responsiveness and influence disease trajectory. Investigating naïve T‐cell responses to controlled polyclonal stimuli provides a means to interrogate these fundamental intrinsic properties while minimizing the confounding effects of antigen history or environmental exposure.

CeD presents a particularly tractable model to interrogate intrinsic T‐cell dysfunction. Unlike many autoimmune disorders managed with immunosuppressive medications, CeD is treated solely through dietary antigen exclusion via strict adherence to a gluten‐free (GF) diet. This offers a unique opportunity to dissect intrinsic differences in fundamental T‐cell response processes in an autoimmune setting without the confounding effects of immunosuppression, which can alter cell survival and perturb signaling and response thresholds.^16^ Moreover, individuals with treated CeD (on a GF diet) and those with active, newly diagnosed disease can be studied in parallel, enabling direct comparisons across antigen exposure and inflammatory states. The persistence of immune phenotypes across these conditions would support a stable, intrinsic component rather than a purely environmentally driven effect.

To address this, we developed a high‐resolution functional profiling platform, the *T cell momentum assay* that enables detailed analysis of lymphocyte fate decisions under defined, controlled conditions. In this assay, polyclonal naïve T cells are subjected to strong, non‐specific stimulation for 42 h, after which the stimulus is withdrawn. The subsequent short burst of proliferation, survival and phenotype change is monitored as a measure of how effectively T cells integrate and respond to activation signals within a defined temporal window. Importantly, the assay controls for changing levels of IL‐2, a major confounding variable in conventional T cell in vitro assays, thereby enabling isolation of intrinsic differences in T cell responsiveness. The response conforms to the Cyton2 model,^17^ allowing quantification of key parameters such as time to first cell division, division destiny (DD), and time to death, while capturing the stochastic nature of lymphocyte fate decisions through probability distribution functions.^18^, ^19^, ^20^, ^21^, ^22^, ^23^

While previous studies using murine in vitro systems and division‐tracking dyes such as Cell Trace Violet (CTV) have provided key insights into the molecular regulation of T‐cell proliferation and survival,^24^, ^25^, ^26^, ^27^, ^28^, ^29^ the T cell momentum assay offers a novel application of these principles to human naïve T cells. Thus, we measure how well T cells accumulate signals in a short period by measuring their proliferation and survival ‘momentum’ after stimulus removal. Using this approach, we compared proliferative and survival responses of polyclonal naive CD4^+^ and CD8^+^ T cells from individuals with CeD and healthy donors (HDs). We identified significant differences in the naïve CD4^+^ T cell compartment of individuals with CeD, including altered IL‐2 secretion and CD69 expression. These findings suggest that naive CD4^+^ T cells in individuals with CeD are functionally distinct at baseline, implicating inherited molecular programming in predisposing the immune system toward dysregulated activation prior to antigen encounter.

## RESULTS

### Development of the human naïve T cell momentum assay

**Figure 1:**
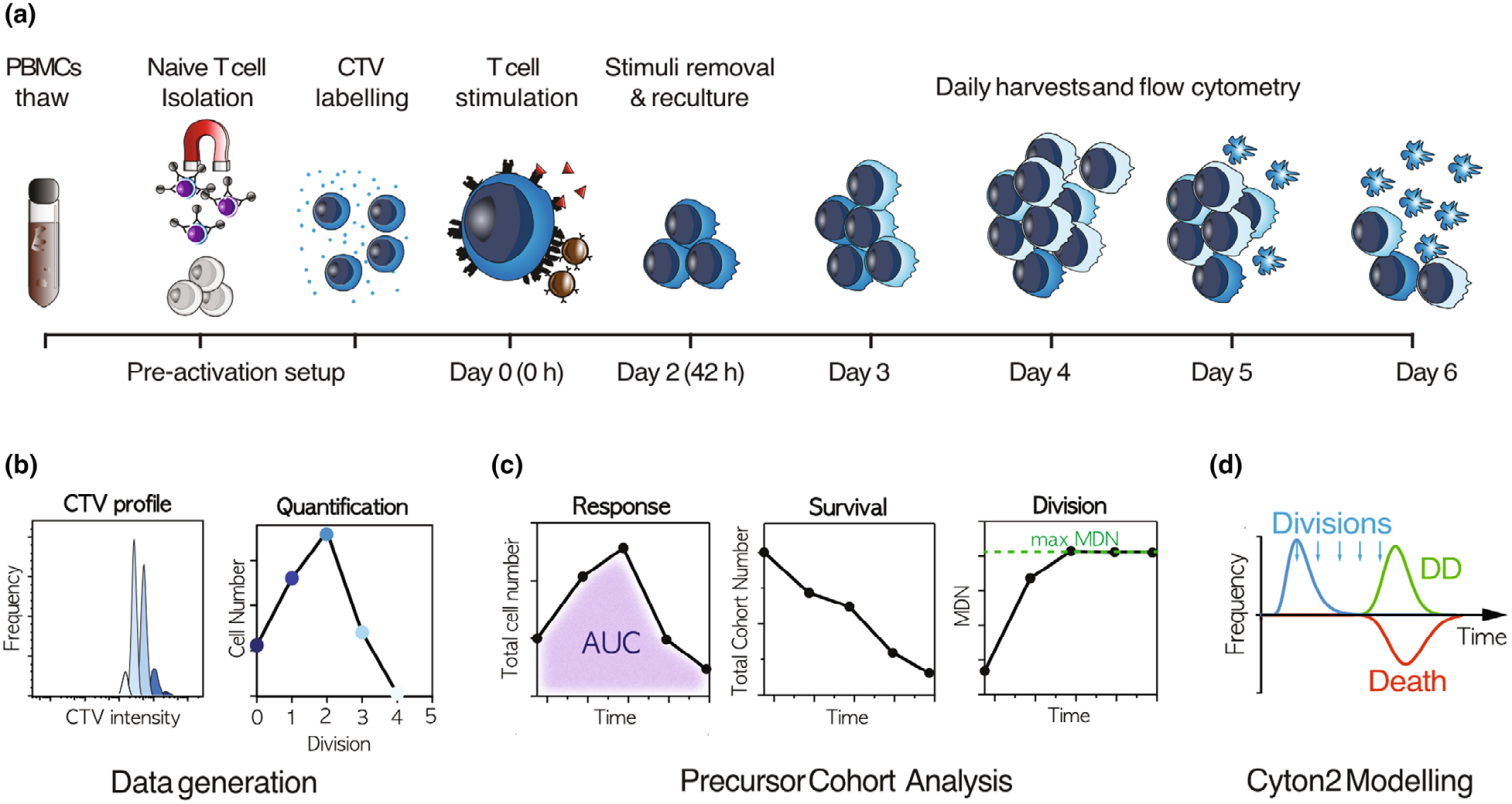
A functional assay to assess division momentum and fate programming in human naïve T cells. **(a)** Overview of the human naïve T cell momentum assay. Cryopreserved PBMCs from HD and CeD individuals were thawed and naïve CD4^+^ or CD8^+^ T cells isolated via negative isolation. Cells were labeled with division‐tracking dye, Cell trace violet (CTV) and stimulated with anti−CD3/CD28 Dynabeads and 100 U/mL rhuIL‐2. After 42 h, activated T cells were washed (and beads magnetically detached) to remove activation signals and recultured for an additional 4 days in media alone or in the presence of blocking IL‐2 conditions (anti‐IL‐2/IL‐2Rα) with or without rhuIL‐2 (31.6 U/mL). Cells were harvested daily and analyzed using flow cytometry. **(b)** Quantitative metrics extracted from the assay. Proliferation was measured by analyzing CTV dilution profiles and calculating precursor cohort numbers within each division generation. **(c)** Precursor cohort analysis enabled quantification: Total cell number to determine overall response magnitude; total cohort number, reflecting survival of the initial input population over time; and mean division number (MDN), for quantification of average division of cell population. Area under the curve (AUC) of total cell numbers over time was used to derive a single proliferative value. Maximum MDN was used as a summary metric reflective of the overall division burst. **(d)** Cell fate timer distributions (time to first division, time to death and time to division destiny, DD) were estimated using the Cyton2 mathematical model, with each modeled as a lognormally distributed random variable.

To quantify proliferation, division and survival of activated naïve CD4^+^ and CD8^+^ T cells from HD and CeD individuals, we developed a novel ‘momentum assay’. Conventional T cell stimulation assays that allow endogenous IL‐2 secretion are highly sensitive to initial cell density, making quantitative comparisons difficult. To address this, we designed an assay to probe the programmed proliferative potential accumulated by activation of human naïve T cells with anti‐CD3, anti‐CD28 and exogenous IL‐2 for 42 h, followed by withdrawal of stimulation to track proliferation, division and survival over four additional days (Figure 1a).

Naïve CD4^+^ and CD8^+^ T cells were isolated and labeled with CTV, achieving ~95–96% purity (Supplementary figure 1a). CTV‐labeled cells were activated with anti‐CD3/anti‐CD28‐coated Dynabeads and recombinant human IL‐2 (rhuIL‐2) for 42 h to ensure maximal activation. T‐cell responses, including survival and division during the stimulation period (0–42 h) were measured prior to stimulus removal. After 42 h, activation signals were withdrawn, and cells were extensively washed and resuspended in fresh media to minimize cytokine carryover prior to reculture. Cells were then cultured under three conditions. First, washed cells were cultured in fresh media alone to measure proliferative momentum programmed during the initial 42 h stimulation period. This culture is also affected by any endogenous IL‐2 produced by the activated cells. Second, cells were recultured in the presence of anti‐IL‐2/anti‐IL‐2Rα blocking antibodies to reduce IL‐2 availability and inhibit signaling through the high affinity IL‐2 receptor (Supplementary figure 2d). At the concentrations used, this combination effectively neutralized IL‐2 at levels substantially exceeding those expected to be produced in these cultures (up to 10 U/mL, or equivalent of 1 ng/mL). These conditions therefore constrain IL‐2 signaling to a low and functionally comparable range across samples. In the third condition, rhuIL‐2 (31.6 U/mL) was added in the presence of IL‐2/IL‐2Rα blockade (Supplementary figure 2e). Under these conditions, IL‐2 preferentially improved cell survival without promoting further division when the high affinity receptor was inhibited (Supplementary figure 2f–h). This feature was used to stabilize later division cohort numbers and enabled more reliable quantification of mean division number (MDN) at later timepoints.

Cell division was monitored daily by flow cytometry (Supplementary figure 1b), and total cell counts per division were quantified (Figure 1b). Summary statistics such as total cell numbers, total cohort numbers and MDN over time were derived using the precursor cohort method,^18^, ^27^, ^30^ which provides interpretable metrics reflecting response magnitude, population survival and division progression, respectively (Figure 1c). The area under the curve (AUC) of normalized total cell counts was also computed as an aggregate measure of proliferative output. To gain mechanistic insight into the cell population dynamics underpinning these responses, we employed the Cyton2^17^ model, which estimates parameters including three stochastic variables: time to first division, time to division destiny (DD), time to cell death, alongside a constant for subsequent division intervals (Figure 1d). The model assumes lognormal distributions for these variables and provides an excellent fit to the experimental data.

We validated the assay robustness across different cell densities (both at the initial culture and after reculture) and Dynabead ratios (Supplementary figure 2a–c). Addition of anti‐IL‐2 and anti‐IL2Rα with rhuIL‐2 (31.6 U/mL) enhanced cell survival without affecting division (Supplementary figure 2e–h), and expression levels of the division regulator, Myc^24^ declined similarly across all conditions (Supplementary figure 2i). Initial analysis in healthy donors showed no strong effect of donor sex or age on overall CD4^+^ or CD8^+^ T‐cell responses (Supplementary figures 3–5), although further stratified analyses are presented below. Having validated the momentum assay in healthy donors, we next applied it to our CeD cohort in parallel to evaluate naïve T‐cell responses and identify disease‐associated differences.

### Early activation responses reveal enhanced early survival in CeD CD8 ^+^ T cells during activation

**Figure 2:**
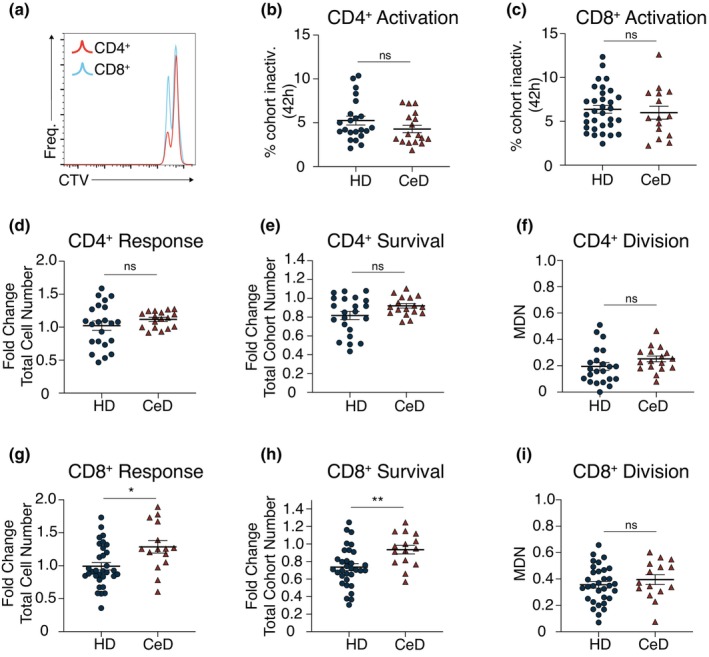
Enhanced early survival of naive CD8^+^ T cells from CeD donors prior to stimulation removal. Naïve CD4^+^ and CD8^+^ T cells from healthy donors (HD) and individuals with Coeliac Disease (CeD) were stimulated using the momentum assay: stimulated for 42 h with anti−CD3/CD28 Dynabeads and 100 U/mL rhuIL‐2. **(a)** Representative CTV proliferation profiles from activated naïve CD4^+^ and CD8^+^ T cells after 42 h. **(b, c)** Frequencies of CD4^+^ (b) and CD8^+^ (c) T cells were measured based on CTV dilution and size as an estimate of T‐cell activation. **(d–f)** Quantification of 42‐h CD4^+^ T‐cell response from HD and CeD donors: (d) total cell number (overall response), (e) total cohort number (survival) and (f) mean division number (MDN; proliferation). **(g–i)** Quantification of 42‐h CD8^+^ T‐cell response: (g) total cell number, (h) total cohort number and (i) MDN. Data are presented as fold‐change relative to matched unstimulated controls. Mean ± SEM for HD and CeD groups. Sample sizes: CD4^+^ T cells (HD = 22, CeD = 17); CD8^+^ T cells (HD = 32, CeD = 15). Comparisons performed using unpaired *t*‐test with Welch's correction. *indicates *P* < 0.05; ** indicates *P* < 0.01.

To assess early activation responses, purified CTV‐labeled naive CD4^+^ or CD8^+^ T cells from HD and CeD donors were stimulated for 42 h with anti‐CD3/anti‐CD28 Dynabeads in the presence of 100 U/mL rhuIL‐2. Both cell types began to divide within this period (Figure 2a), and the percentage of small, undivided cells, resembling inactive (non‐activated) cells (Supplementary figure 1b) was low (~5–6%) across all donors (Figure 2b, c). No significant differences were observed in total cell numbers, total cohort numbers (indicative of cell survival) or MDN between HD and CeD samples for CD4^+^ T cells (Figure 2d–f). In contrast, CD8^+^ T cells from CeD donors showed increased total cell and cohort numbers compared to HD, while MDN remained comparable in the full dataset (Figure 2g–i), indicating enhanced survival with no clear difference in division at this stage. Together, these findings indicate that naïve CD4^+^ T cells from CeD donors undergo normal early activation, whereas CD8^+^ T cells exhibit modestly enhanced survival during the initial activation phase.

To explore whether active inflammatory status in CeD influenced early CD4^+^ responses, we stratified CeD donors based on disease status: those following a gluten‐free (GF) diet and those with newly diagnosed active CeD still consuming gluten (Supplementary figure 6a). CD4^+^ T cells from donors with active CeD exhibited a small but significant increase in total cell numbers and survival compared to treated CeD donors and healthy controls; however, no difference was observed in activation or division. These findings suggest that disease state had a small underlying effect on early cell survival within CD4^+^ T cells from active but not treated CeD individuals. Together, early kinetic analysis confirms that naive CD4^+^ and CD8^+^ T cells from both HD and CeD donors undergo robust activation within 42 h. However, CD8^+^ T cells from CeD donors, and to a lesser extent CD4^+^ T cells from active CeD cases, display slightly enhanced survival during this early phase.

### Hypo‐proliferative naïve CD4 ^+^ T‐cell responses in CeD donors

Despite largely preserved early activation, we next asked whether differences in proliferative behavior programmed during the initial stimulation period became apparent following stimulus withdrawal. We therefore assessed the proliferative momentum of naive T cells from HD and CeD donors following stimuli withdrawal at 42 h.

**Figure 3:**
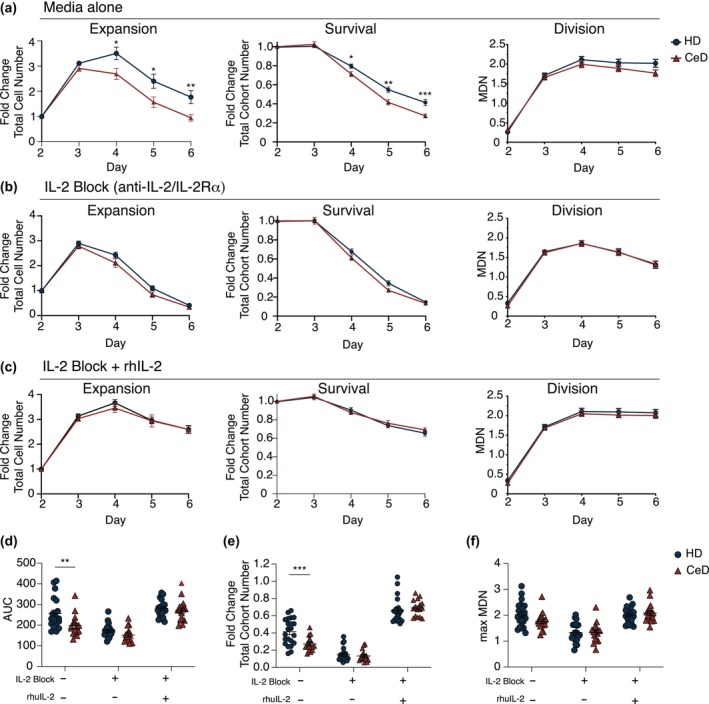
Hypoproliferation of naïve CD4^+^ T cells from CeD donors is IL‐2 dependent. Naïve CD4^+^ T cells from HD and CeD donors were stimulated for 42 h with anti−CD3/CD28 Dynabeads and 100 U/mL rhuIL‐2, followed by stimulus removal. Cells were cultured for an additional 4 days in media alone or with IL‐2 blocking conditions and harvested daily for flow cytometric analysis. **(a)** CD4^+^ T cells recultured in media alone: fold change total in (left) total cell number, (middle) total cohort number (survival) and (right) MDN (proliferation). **(b)** CD4^+^ T cells cultured with anti‐IL‐2/IL‐2R**α** antibodies: (left) total cell number, (middle) total cohort number and (right) MDN. **(c)** CD4^+^ T cells cultured with anti‐IL‐2/IL‐2R**α** antibodies plus exogenous rhuIL‐2: (left) total cell number, (middle) cohort number and (right) MDN. **(d–f)** Summary measures across conditions (d): area under the curve (AUC) for total cell number, (e) fold‐change in total cohort number at day 6 and (f) maximum MDN. Data shown as mean ± SEM; sample sizes: HD = 22, CeD = 16. Comparisons were made using unpaired *t*‐test with Welch's correction. ** indicates *P* < 0.01; *** indicates *P* < 0.001.

When recultured in media‐alone, CD4^+^ T cells from CeD donors exhibited significantly lower total cell numbers from day 4 onwards (Figure 3a). This hypoproliferation stemmed from reduced survival, not division rate, as division kinetics and MDN were mostly comparable.

Given the skewed sex distribution within the CeD cohort, we assessed if sex influenced CD4^+^ T‐cell responses. Initial analysis stratified by sex showed no consistent differences in response magnitude or kinetics between male and female donors (Supplementary figure 3). To further control for potential confounding by sex, we repeated the analysis in female‐only subsets (HD vs CeD; *n* = 10, *n* = 15, respectively), which preserved effect sizes and statistical significance across key metrics (Supplementary figure 4), supporting that the observed CD4^+^ T cell hypoproliferation phenotype is not driven by sex bias. No differences were observed between active and treated CeD groups (Supplementary figure 6a, b) and non‐activated cells from both groups showed similar rates of cell death (Supplementary figure 6c, d), indicating that survival differences were specific to the activated state.

Blocking IL‐2 through the addition of anti‐IL‐2Rα/IL‐2 antibodies reduced survival differences and normalized expansion kinetics between CeD and HD groups (Figure 3b). Moreover, supplementing IL‐2 in the presence of IL‐2 blockade restored survival in both groups without altering the division parameters (Figure 3c). AUC analysis of total cell numbers confirmed reduced proliferative output in CeD samples only in the media‐alone cultures, but not when IL‐2 signaling was neutralized or normalized through addition of excess levels of IL‐2 (Figure 3d,e). MDN remained unchanged across all conditions (Figure 3f), implicating impaired IL‐2‐mediated survival rather than division defects. In contrast, CD8^+^ T cells from CeD showed no IL‐2‐dependent differences in expansion kinetics (Supplementary figure 7), despite evidence of altered early activation and proliferative dynamics in the first 42 h of stimulation, highlighting a selective CD4^+^ T cell dysregulated response upon activation, characterized by hypoproliferation driven by impaired IL‐2 dependent survival.

### Cyton2 modeling reveals early death timers in CD4 ^+^ T‐cell responses from CeD donors

**Figure 4:**
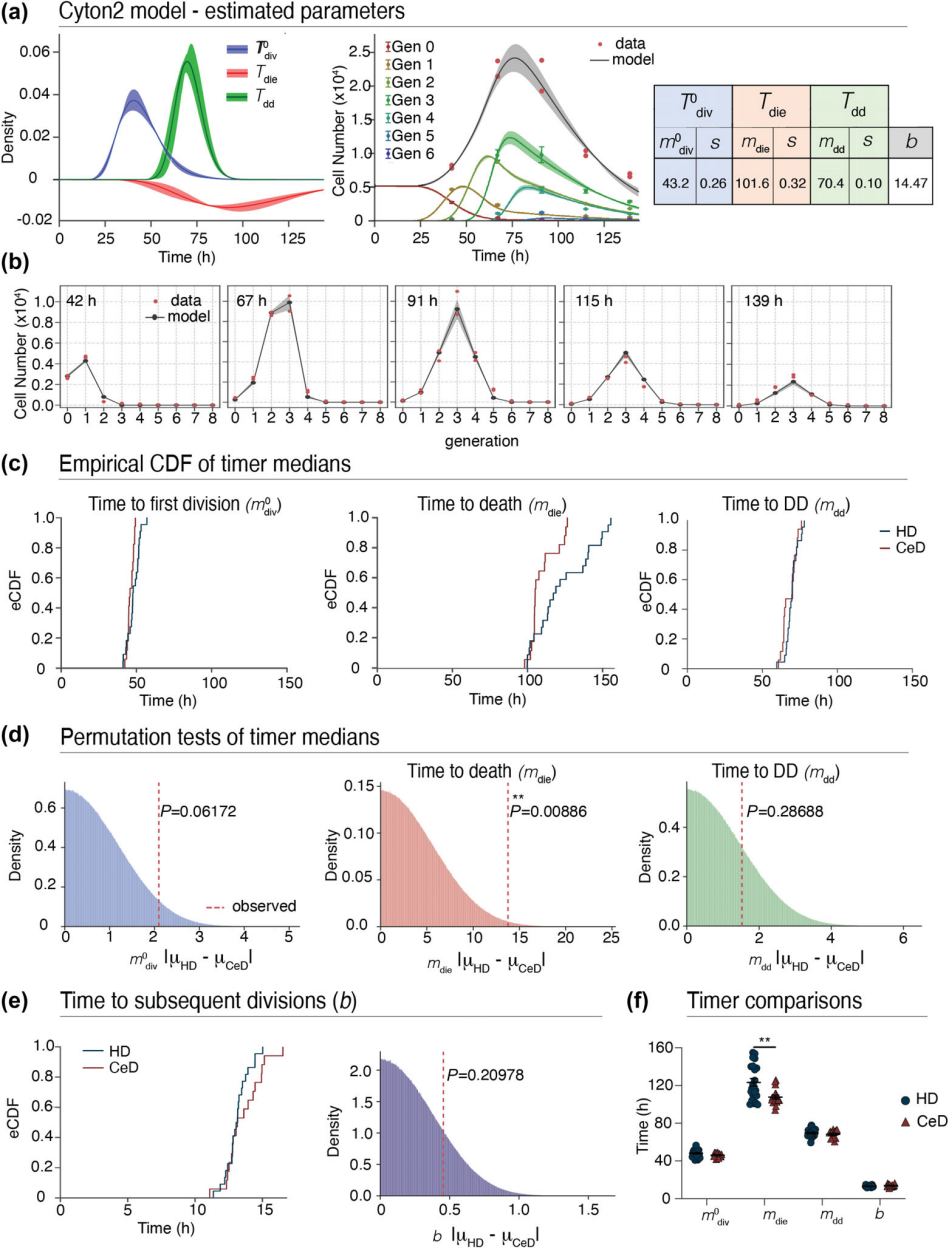
Cyton2 modeling reveals earlier cell death in CD4^+^ responses from CeD donors. Cyton modeling was applied to CD4^+^ T cell momentum assay data to estimate underlying cell fate timers. **(a)** Example best‐fitted Cyton2 parameters showing lognormal distributions (±95% CI) for time to first division (T^0^_div_), time to die (T_die_) and time to DD (T_dd_; left panel). Model extrapolation of total cell numbers in generations 0–8 over time (middle). Estimated median (m) and log‐variance (s) for T^0^_div_, T_die_, T_dd_ and a constant for subsequent division time (b) for represented donor sample (right). **(b)** Model fit to observed cell number per generation for each time point. **(c)** Empirical cumulative distribution functions (eCDFs) comparing HD and CeD: median time to first division ($m_{\mathrm{div}}^{0}$), median time to die ($m_{\mathrm{die}}$) and median time to DD (m_dd_), as indicated. **(d)** Permutation testing comparing observed differences between average HD and CeD medians (red dashed line) for: $m_{\mathrm{div}}^{0}$, $m_{\mathrm{die}}$ and $m_{\mathrm{dd}}$. **(e)** eCDF comparing subsequent division constant (*b*) between HD and CeD cohorts. Permutation test comparing average subsequent division (*b*) between HD (black) and CeD (red) cohorts. **(f)** Summary comparison of medians model parameters ($m_{\mathrm{div}}^{0}$, $m_{\mathrm{die}}$, $m_{\mathrm{dd}}$) and subsequent division constant (*b*) between groups. Data are presented as means of timer medians and *b* ± SEM; (HD = 22 CeD = 16). Comparisons used unpaired *t*‐tests with Welch's correction.

To gain mechanistic insight into the hypo‐proliferative phenotype observed in CD4^+^ T cells from CeD donors, we applied the Cyton2 model to division data from cells cultured in media alone (from Figure 3a). This model allows estimation of stochastic cellular fate parameters—specifically, the medians and log‐variances of the distributions for timers of first division (T^0^_div_), death (T_die_) and division destiny (T_dd_), as well as the average time between subsequent divisions (parameter *b*). Best‐fit model and parameter values and 95% confidence intervals were obtained using least‐squares fitting and bootstrap methods, respectively (Figure 4a). The model was fitted to observed total cell numbers per generation over time across both HD and CeD cohorts (Figure 4b). Model fits for all samples were well constrained for all parameters, supporting the suitability of Cyton2 for these data (see https://github.com/hodgkinlab/CeDmomentum2025). Empirical cumulative distribution functions (eCDFs) were generated for each fate timer using cohort‐specific median (*m*) values. This analysis revealed that CD4^+^ T cells from CeD donors exhibited significantly earlier death times (m_die_) compared to HD, while time to first division ($m_{\mathrm{div}}^{0}$) and division destiny times (m_dd_) were comparable between groups (Figure 4c). To test the significance of these differences, we performed hypothesis testing using a non‐parametric permutation test. Here, the observed difference in medians between HD and CeD cohorts was compared to a null distribution generated from 10^7^ random permutations. This confirmed that the earlier death time in CeD (time ± CI) was statistically significant, while differences in m^0^_div_ and m_dd_ were not (Figure 4d).

We further assessed the average time for subsequent divisions (*b*) and found no significant differences between cohorts (Figure 4e). This was confirmed using *t*‐tests on cohort level medians for each timer and subsequent division, which again demonstrated a significant reduction in m_die_ for CeD donor samples, but no differences in $m_{\mathrm{div}}^{0}$, m_dd_ or *b* compared to HD (Figure 4f). Finally, we examined whether IL‐2 signaling accounted for any observed differences in cellular timer distributions. When Cyton2 modeling was applied to cultures in which IL‐2 was neutralized or supplemented (as in Figure 3), no differences in timer medians were observed between HD and CeD cohorts for either CD4^+^ or CD8^+^ T‐cell responses (Supplementary figure 8). Together, Cyton2 modeling provides quantitative supporting evidence that the impaired proliferative capacity of CD4^+^ T cells from CeD donors is driven by altered cell fate dynamics in cultures lacking control of endogenously produced IL‐2.

### Activated naïve CD4 ^+^ T cells from CeD donors secrete less IL‐2, but express normal CD25

**Figure 5:**
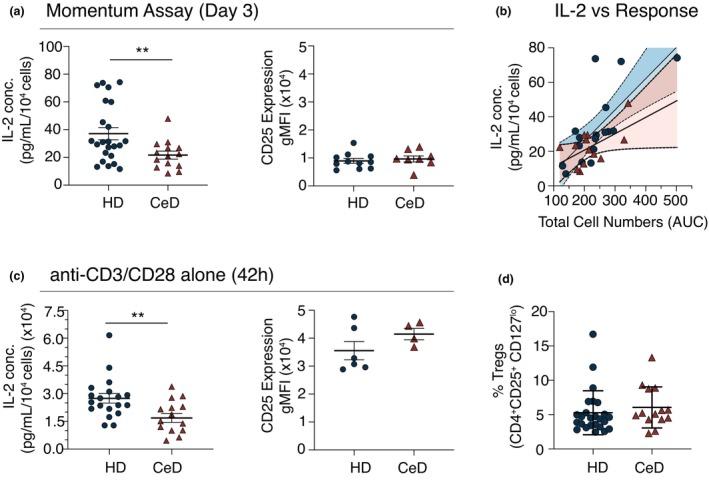
Reduced IL‐2 secretion contributes to hypo‐proliferative responses of activated naïve CD4^+^ T cells from CeD donors. Naïve CD4^+^ T cells from HD and individuals with CeD were stimulated using the momentum assay and IL‐2 production and IL‐2R**α** expression were assessed following activation. **(a)** IL‐2 concentration was measured in supernatants collected at day 3 of the momentum assay from CD4^+^ T cell cultures originally stimulated with αCD3/CD28 Dynabeads in the presence of IL‐2 for 42 h (HD = 23, CeD = 14). Surface expression of CD25, the high affinity IL‐2 receptor alpha chain, was quantified by flow cytometry (HD = 6, CeD = 4). **(b)** Correlation between IL‐2 secretion (pg/ml/10^4^ cells) and cumulative proliferation (total cell number AUC) in momentum assay across HD and CeD donors, with linear regression equations: HD, Y = 0.18X − 10.38; CeD, Y = 0.11X – 2.82. **(c)** To assess early IL‐2 production, supernatants and cells were collected at 42 h from cultures containing anti‐CD3/CD28 Dynabeads alone. IL‐2 concentration (left; HD = 19, CeD = 14) and CD25 expression (right; HD = 11, CeD = 8) on CD4^+^ T cells were quantified. **(d)** The frequency of circulating Tregs (CD4^+^ CD25^+^ CD127^−^) was measured ex vivo in PBMCs (HD = 25; CeD = 14) as an overall percentage of T cells. Data are presented as mean values ± SEM. Statistical comparisons were performed using unpaired *t*‐test with Welch's correction. Linear regression was used to compare slopes and intercepts in (b). ** indicates *P* < 0.01.

Given the impaired IL‐2‐dependent survival defect observed in CeD CD4^+^ T cells, we next investigated whether changes in IL‐2 secretion or expression of the high‐affinity IL‐2Rα (CD25) could explain the hypo‐proliferative responses seen in CeD donors. On day 3 of the momentum assay, IL‐2 levels were significantly lower in cultures from CeD donors compared to HD, while CD25 expression was comparable between groups (Figure 5a). IL‐2 concentration in supernatants significantly correlated (HD *P* < 0.01, CeD *P* < 0.05) with total cell number AUC for both groups (Figure 5b), and regression slopes were similar (Figure 5b), indicating preserved IL‐2 responsiveness.

To validate, we restimulated CD4^+^ T cells without exogenous rhuIL‐2 for 42 h and again found endogenous IL‐2 secretion was significantly reduced in CeD donors compared to HD, with normal CD25 expression (Figure 5c). In contrast, CD8^+^ T cells showed no differences in IL‐2 secretion or CD25 expression between HD and CeD cohorts (Supplementary figure 9a–d). Disease state stratification revealed no significant IL‐2 differences (Supplementary figure 9e, f), suggesting this defect is stable regardless of gluten exposure. Together, these data demonstrate that reduced IL‐2 secretion by activated naïve CD4^+^ T cells in CeD underpins their impaired proliferative capacity.

We next addressed whether this reduction in IL‐2 might also impact T regulatory (Treg) cell frequencies, as these cells require IL‐2 for survival and homeostasis, as a potential driver for disease in CeD. The proportion of circulating Tregs (CD3^+^CD4^+^CD25^+^CD127^−^ cells) was determined in PBMCs from CeD (*n* = 14) and HD (*n* = 25) (Supplementary figure 10a). Similar proportions of Tregs in the CD4^+^ T cell pool were observed between HD and CeD cohorts (Figure 5d), indicating no overt reduction in circulating Tregs.

### Delayed CD69 downregulation in CeD CD4 ^+^ T cells

**Figure 6:**
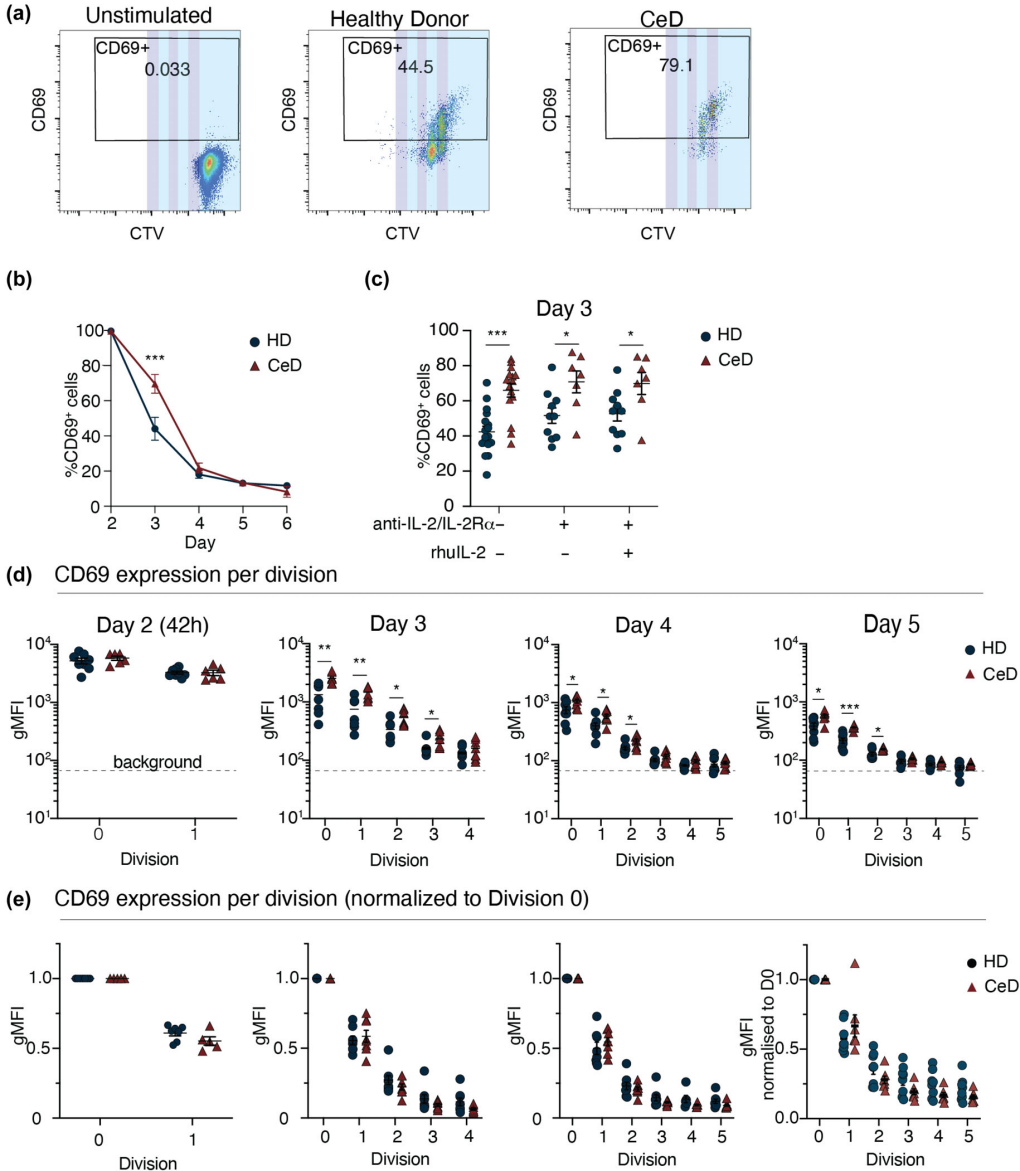
CD69 remains elevated after stimuli removal then declines at a normal rate in naïve CD4^+^ T cell responses from CeD donors. Surface expression of early T‐cell activation marker CD69 was measured on CD4^+^ T cells from HD and CeD individuals during the momentum assay. CD69^+^ cells and division‐based kinetics were determined as shown using **(a)** Representative plots of division‐linked CD69 expression in HD, CeD and unstimulated controls, with gating strategy shown. **(b)** Percentage of CD69^+^ (% total CD4^+^ T cells over time following activation and stimulus withdrawal HD, *N* = 8; CeD, *N* = 8). **(c)** CD69 expression was quantified at day 3 post activation in cells cultured under three conditions: media alone (HD, *N* = 16; CeD, *N* = 14), anti‐IL‐2/IL‐2R**α** blockade (HD, *N* = 8; CeD, *N* = 7) and anti‐IL‐2/IL‐2Rα blockade with exogenous rhuIL‐2 supplementation (HD, *N* = 8; CeD, *N* = 7). **(d)** CD69 gMFI was plotted against division number to assess activation kinetics in dividing cells. **(e)** Fold change CD69 gMFI relative to division 0 across divisions is shown for day 2, 3 and 4 post activation, as indicated (HD and CeD, *N* = 8 each). Data are presented as mean ± SEM. Statistical comparisons were performed using unpaired t‐tests with Welch's correction. * indicates *P* < 0.05; ** indicates *P* < 0.01; *** indicates *P* < 0.001.

To explore possible signal integration defects in CeD CD4^+^ T cells, we assessed CD69 expression, an early activation marker and downstream target of TCR signaling, following stimulus removal. CTV‐labeled CD4^+^ T cells were stimulated with anti‐CD3/CD28 beads and rhuIL‐2 for 42 h as previously, after which the stimuli were washed off and CD69 expression was measured over time and by division (Figure 6a, b, Supplementary figure 10b). Interestingly, while CD69 levels were comparable immediately after stimulation, CeD CD4^+^ T cells retained higher CD69 expression 24 h later (day 3), independent of IL‐2 availability. Elevated CD69 expression was restricted to early division generations (generations 0–3 on day 3, 0–2 on days 4 and 5; Figure 6d). However, when expression was normalized to undivided cells (generation 0) levels within each timepoint, no differences were observed between groups (Figure 6e), suggesting this observed pattern is consistent with delayed downregulation of activation signals rather than increased activation per se. Other activation markers, including CD62L and CD127, were similar between HD and CeD donors (Supplementary figure 10c). Within CD8^+^ T cells, CD69 levels were similar between HD and CeD cohorts (Supplementary figure 10d, left panel). Interestingly, CD69 on CD4^+^ T cells was significantly higher in individuals with active CeD compared to those on a GF diet. However, both groups showed significantly elevated expression relative to HD controls (Supplementary figure 10d right panel), suggesting that active disease may exacerbate, but is not solely responsible for, this phenomenon. Collectively, these findings reveal that activated naïve CD4^+^ T cells from CeD donors exhibit delayed downregulation of CD69 following stimulus withdrawal, further supporting a model of dysregulated early signal integration and activation in CeD.

## DISCUSSION

Using our novel T cell momentum assay, we identified functional dysregulation in polyclonal naïve T cells from individuals with coeliac disease (CeD), most notably in CD4^+^ T cells. While naïve CD8^+^ T cells from CeD donors exhibited a modest early survival advantage and altered early activation dynamics at 42 h post‐stimulation, CD4^+^ T cells displayed a marked hypo‐proliferative response after withdrawal of exogenous stimuli. Cyton2 modeling revealed this impaired proliferation momentum was primarily driven by accelerated cell death, marked by significantly shortened death timers. Importantly, parameters such as time to first division and DD timers as well as survival of unstimulated cells remained unaffected, indicating that dysregulated cell death is a specific and defining feature of CD4^+^ T cells in CeD. Mechanistically, reduced IL‐2 secretion emerged as a key driver of this impaired survival. Notably, defects in the naïve T cell compartment have not previously been described in CeD. These findings raise important questions about the origin and role of naïve T‐cell dysfunction in CeD and its potential contribution to disease development, progression and prognosis.

IL‐2 is critical for T cell proliferation and survival,^19^ yet our data demonstrate that reduced IL‐2 secretion by CD4^+^ T cells in CeD selectively compromises cell survival with minimal impact on division timing. Notably, CD25 expression was comparable between groups, whereas CeD CD4^+^ T cells produced less IL‐2 under identical stimulation conditions, indicating reduced cytokine availability as the primary driver of impaired survival. Measured IL‐2 concentrations reflect the balance of cytokine secretion and consumption within the culture system and therefore represent net IL‐2 availability rather than secretion alone. Consistent with this, the survival advantage observed for HD cells was abrogated, and survival in both groups equalized when IL‐2 signaling through the high‐affinity IL‐2 receptor was controlled via addition of anti‐IL‐2/IL‐2Rα blockade or supplemented with high levels of rhuIL‐2. This selective sensitivity may reflect altered utilization of IL‐2 signaling pathways, potentially involving low affinity IL‐2 receptor components (IL‐2Rγ and β chains) that favor pro‐survival over pro‐proliferative responses. Such mechanisms may contribute to the phenotype observed in CeD‐derived CD4^+^ T cells following stimulus withdrawal, where low IL‐2 availability biases survival dynamics. The molecular basis for differential IL‐2 signal integration remains unclear and warrants further mechanistic investigation. Nonetheless, our findings highlight reduced IL‐2 secretion by CD4^+^ T cells as a defining and functionally relevant feature of naïve CD4^+^ T cells in CeD, consistent with dysregulated integration of TCR and costimulatory signals at the molecular level.

We also observed aberrant CD69 kinetics in CeD CD4^+^ T cells, with delayed downregulation after stimulus withdrawal. As CD69 is typically downregulated following TCR signal termination,^31^ this delay suggests impaired negative regulation of activation that normally constrains T‐cell activation dynamics. While heightened sensitivity to TCR stimulation and increased TCR affinity for self‐antigens have been implicated in the pathogenesis of autoimmune disorders,^32^ our quantitative momentum assay, specifically designed to assess intrinsic TCR responsiveness, revealed no evidence of intrinsic TCR hypersensitivity in CeD naïve CD4^+^ T cells.

Together, these findings reveal a previously unrecognized functional defect in the naïve CD4^+^ T cell compartment in CeD, marked by impaired survival, reduced IL‐2 secretion and delayed TCR deactivation. This immune phenotype may represent an early functional risk state that contributes to disease susceptibility or progression and has important implications for understanding immune dysregulation underlying CeD, and autoimmune conditions, more broadly.

The proliferative differences observed in CD4^+^ T‐cell responses from CeD donors reflect an impaired ability of these cells to integrate stimuli. This dysfunction likely stems from cell‐intrinsic defects in the sensing and interpretation of stimulatory inputs, potentially involving abnormal receptor and/or downstream signaling processes. Indeed, TCR signaling via CD3 and costimulatory receptor CD28 converges on pathways that activate key transcription factors driving IL‐2 production.^33^, ^34^ Aberrant signaling may therefore arise from dysregulated receptor function, defects in downstream signaling proteins, or impaired transcriptional responses ‐ alone or in combination. Supporting this hypothesis, genome‐wide association studies (GWAS) have linked CeD risk to variants in genes involved in T‐cell activation, including loci within the IL‐2 and associated signaling pathways.^11^, ^12^, ^14^ Further, transcriptomic analyses of bulk CD4^+^ T cell populations from CeD patients have similarly revealed differential expression of genes involved in T‐cell activation, cytokine signaling and other critical immuno‐molecular pathways.^31^, ^35^

A key question is if these observed differences reflect genetically encoded immune programming or the cumulative effects of prior environmental or inflammatory exposure. While these factors cannot be fully disentangled in human studies, several observations support a substantial intrinsic component. First, the phenotype is evident in naïve T cells prior to antigen‐driven differentiation. Second, it is preserved in individuals on a gluten‐free diet, arguing against dependence on ongoing antigen exposure. Third, the affected pathways align closely with known genetic risk loci in CeD.^10^, ^11^, ^12^, ^13^, ^14^ Together, these findings are consistent with a model in which polygenic variation shapes baseline T‐cell functional setpoints, although environmental influences may further modulate these responses.

It is plausible that this naïve T cell dysfunction in CeD arises early, driven by germline polymorphisms affecting T‐cell activation or cytokine production, and may be further shaped by epigenetic modifications influenced by environmental exposures, such as infections or diet. Longitudinal birth cohort studies of genetically at‐risk infants will be critical to determine whether these naïve T cell abnormalities are present at birth or emerge later prior to clinical onset of CeD. Predictive tools such as genomic risk scores^36^, ^37^ and disease‐specific proteomic signatures in blood^38^ both demonstrate a potential for identifying individuals likely to develop CeD, further supporting the hypothesis that genetically‐determined immune dysfunction is a likely precursor to overt CeD development.

A lowered capacity for IL‐2 production may be a key mechanism linking naïve CD4^+^ T cell dysfunction to breakdown of tolerance and disease development. Insufficient IL‐2, whether due to limited availability or receptor defects, has been implicated in various autoimmune and autoinflammatory conditions.^39^, ^40^, ^41^ This is particularly relevant for Tregs, which rely on adequate IL‐2 for development, expansion and suppressive function.^42^ If naïve CD4^+^ T cells in CeD are intrinsically impaired in their ability to produce IL‐2, this may compromise Treg homeostasis and promote autoimmunity. Whether reduced IL‐2 is an early, consistent feature in genetically at‐risk individuals, and whether it precedes or predicts disease onset, remains an open and important question. These findings contrast with robust IL‐2 production observed in treated CeD patients following oral gluten challenge, where circulating IL‐2 levels can exceed 100 pg/mL within 2–4 h of gluten ingestion and remain elevated for up to 6 h.^3^, ^43^ In that setting, IL‐2 is secreted by gluten‐specific memory CD4^+^ T‐cells and likely supports the expansion of these pathogenic effector cells. Given that treated CeD patients often experience small, unintentional gluten exposures (typically < 1–2 g), it is plausible that this leads to intermittent, low‐level IL‐2 release. Such episodic IL‐2 release could influence the broader T cell compartment, including naïve T cells. Whether such episodic IL‐2 exposure contributes to altered homeostatic programming of naïve T cells over time warrants further investigation.

CeD shares several immunogenetic features with other autoimmune conditions, including Type 1 diabetes, rheumatoid arthritis, multiple sclerosis and autoimmune thyroid disease. Many of these conditions involve overlapping genetic risk loci that affect IL‐2 signaling, T‐cell activation and co‐stimulation, and cytokine response pathways.^35^, ^44^, ^45^ The high prevalence of autoimmune comorbidities among individuals with CeD further supports the idea of shared underlying defects in immune regulation.^15^, ^46^ In this context, applying the T cell momentum assay to other autoimmune cohorts may help determine whether naïve T‐cell dysregulation represents a generalizable early immune signature of autoimmunity.

Our findings suggest that intrinsic baseline programming of naive CD4^+^ T cells is fundamentally altered in individuals with CeD. These cells exhibit defective integration of activation signals, impaired IL‐2 secretion and reduced proliferative momentum. We predict these underlying genetic programming differences will also influence T cell immune responses initiated in vivo, whether against foreign or autoreactive antigens. The momentum assay was instrumental in resolving these dynamic features and provides a valuable platform for dissecting early functional abnormalities in proliferative timers of division, death and DD in human T cells. Armed with this assay, our findings set the scene to explore whether naïve T‐cell dysfunction represents a preclinical immune phenotype that contributes to CeD and broader autoimmune pathogenesis in at‐risk individuals.

Altogether, we propose a model in which subtle, largely cell‐intrinsic defects in signal integration and fate programming within naïve CD4^+^ T cells establish a permissive immune setpoint in CeD, shaping susceptibility and facilitating the emergence of pathogenic gluten‐specific responses. These alterations, characterized by impaired IL‐2 secretion and prolonged activation, suggest that dysregulated baseline programming within the naïve compartment may precede and influence antigen‐driven pathology. Beyond CeD, the momentum assay offers a scalable, model‐informed platform to quantify early T cell dysregulation in primary human samples, enabling comparative functional profiling across autoimmune disease, polyautoimmunity and immune‐mediated conditions where inherited risk is evident, but functional consequences remain poorly resolved.

## METHODS

### Coeliac and healthy participants

Blood samples from individuals with CeD were obtained through the Coeliac Research Database. 22 CeD participants (median age, 44 years; range, 20–69 years; female, 19 (86%)) donated whole blood. Sixteen participants were on a strict gluten‐free diet for at least 6 months (treated CeD) and 6 were recently diagnosed (active CeD). All CeD diagnoses were confirmed by serology and characteristic duodenal histopathology (Table 1). Fifty‐four healthy donors (HDs; median age, 48 years; range, 21–75 years; female 23, 43%) were included. Eighteen buffy coats were sourced from the Australian Red Cross LifeBlood (ARCLB), and 36 whole blood samples were collected via the WEHI Volunteer Biospecimen Donor Registry (VBDR). Healthy donors self‐reported no immune‐mediated diseases, no immune‐modulatory medication use and no active illness. Peripheral blood mononuclear cells (PBMCs) were isolated from all samples.

**Table 1:** Clinical information, HLA haplotype and demographics of Coeliac Disease participants.

Patient ID	Disease status	Age	Sex	Age Diagnosis	Other AI	Family Disease Hx	Medications	HLA DQ Haplotype (zygosity)	Patient PMHx
P01	Treated	64	F	49	T1DM	Pernicious anemia (mat. grandmother); Hashimoto's disease (mother)	Nil of note	DQ 2.5/8	Nil of note
P02	Treated	61	F	51	Nil Known	Graves' Disease (mother)	Nil of note	DQ 2.5 (Hom)	Nil of Note
P03	Treated	69	F	64	Autoimmune thyroiditis	CeD (1 Brother, 3 nephews); Addison's disease (brother); Rheumatoid arthritis (sister)	Thyroxine/ Antihypertensive	DQ 2.5 (Hom)	Nil of note
P04	Treated	53	M	49	Nil Known	CeD (mother, nephew)	Nil of note	DQ 2.2/2.5	Abnormal LFTs
P05	Treated	66	F	62	Graves' disease	CeD (daughter)	Nil of note	DQ 2.5 (Het)	Nil of note
P06	Treated	51	F	47	Nil Known	Nil Known	Thyroxine 75	DQ 2.5 Het	Participant (mother and sister) have “underactive thyroid”; eczema
P07	Treated	27	F	24	Nil Known	T1DM (brother); CeD (2 sisters)	Nil of note	DQ 2.5/8	Nil of Note
P08	Treated	43	F	33	Nil Known	CeD (mother, son)	Nil of note	DQ 2.2/8	Nil of note
P09	Treated	41	F	40	Nil Known	Nil Known	Nil of note	DQ 2.5 Het	Nil of note
P10	Treated	32	F	23	Nil Known	Nil Known	Nil of note	DQ 2.5/2.2	Haemochromatosis
P11	Treated	56	F	55	Hashimoto's disease	Hashimoto's disease (mother); CeD (cousin)	Thyroxine	DQ 2.5 Het	Nil of note
P12	Treated	42	F	41	Nil Known	CeD (father); Rheumatoid arthritis (father); CeD (aunt, uncle)	Nil of note	DQ 2.5/8	Asthma
P13	Treated	43	F	41	Nil Known	T1DM (uncle)	Nil of note	DQ 2.5 Het	Factor 5 Leiden, Polyarthritis
P14	Treated	66	F	56	Nil Known	CeD (cousin)	Nil of note	DQ 2.5 Het	Nil of note
P15	Treated	63	M	43	Nil Known	CeD (brother, 2 sons, daughter)	Nil of Note	DQ 2.5 Het	Nil of note
P16	Treated	79	F	2	Dermatitis herpetiformis	CeD (2 sisters, 1 niece); T1DM (3 sisters, 1 brother); Hashimoto's disease (2 sisters, 1 niece); Graves' disease (mother)	Anti‐hypertensives	DQ 2.5 Het	Hypertension, atrial fibrillation
P17	Active	57	F	57	Nil Known	CeD (son)	Nil of note	DQ 2.5 Het	Lichen sclerosus
P18	Active	32	F	32	Nil Known	Hypothyroidism (mother)	Nil of note	DQ 2.5 Het	Endometriosis, anxiety
P19	Active	28	F	28	Nil Known	Inflammatory Bowel Disease (mother, aunt)	Nil of note	DQ 2.5 Het	Nil of note
P20	Active	29	F	29	Nil Known	Nil Known	Nil of note	DQ 2.5 Het	Nil of note
P21	Active	36	F	36	Nil Known	CeD (brother)	Nil of note	DQ 2.5 Het	Asthma, rosacea, osteopaenia
P22	Active	47	M	47	Nil Known	CeD (mat. aunt)	Nil of note	DQ 2.5/8	Depression

Abbreviations: AI, autoimmunity; CeD, Coeliac Disease; F, female; Het, Heterozygous; Hom, Homozygous; Hx, history; LFT, Liver function tests; M, male; Mat, maternal; PMHx, past Medical History; T1DM, Type 1 Diabetes Mellitus.

### Human naïve CD4 ^+^ and CD8 ^+^ T lymphocyte culture

PBMCs were isolated using Ficoll‐Paque Plus (Leucosep tubes, Greiner), washed and cryopreserved in FBS with 10% DMSO. Thawed PBMCs were resuspended in T cell medium (TCM; RPMI‐1640 with 10% FBS, HEPES, Pen/strep, GlutaMAX, non‐essential amino acids, sodium pyruvate, 2‐mercaptoethanol and Normocin).

Naive CD4^+^ and naive CD8^+^ T cells were isolated using EasySep kits (STEMCELL technologies). Approximately 800 000 naïve CD4^+^ T cells or 400 000 naïve CD8^+^ T cells were required per assay, typically obtained from 20 to 30 × 10^6^ cryopreserved PBMCs, or 20–40 mL whole blood. Post‐isolation purities were ~ 96% for naïve CD4^+^ T cells (CD3^+^CD20^−^CD4^+^CD8^−^CD45RA^+^CD45RO^−^CD27^+^) and ~ 95% for naïve CD8^+^ T cells (CD3^+^CD20^−^CD4^−^CD8^+^CD45RA^+^CD45RO^−^CD27^+^), verified by flow cytometry (Supplementary table 1; Supplementary figure 1). Isolated naïve T cells were labeled with 5 μM CellTrace Violet (CTV, Invitrogen) in PBS/0.1% BSA for 20 min at 37°C, washed and kept on ice until stimulation.

CTV‐labeled naive CD4^+^ or CD8^+^ T cells were seeded into 96‐well round‐bottom plates in TCM with anti‐CD3/CD28‐coated Dynabeads (Gibco) at a 1:1 cell‐to‐bead ratio with recombinant human IL‐2 (100 U/mL, Peprotech). CD4^+^ T cells were plated at 1 × 10^4^ cells/well for CD4^+^; CD8^+^ at 2 × 10^4^ cells/well. After 42 h at 37°C/5% CO_2_, a subset of cells was harvested for flow cytometry. Remaining cells had beads removed using a magnetic separator, were washed to remove residual rhuIL‐2 and recultured for 4 days under one of three conditions:

- media alone;
- anti‐IL‐2/IL‐2R**α** blockade: 5 μg/mL anti‐human IL‐2 antibody (clone MQ1‐17H12, WEHI monoclonal antibody facility) plus 1 μg/mL anti‐CD25 (Simulect Basiliximab; kindly provided by Novartis, Basel, Switzerland);
- IL‐2 supplementation following blockade: anti‐IL‐2/anti‐CD25 as above plus 31.6 U/mL rhuIL‐2.

Cells were washed twice following bead removal to minimize residual cytokine and stimulation carryover prior to reculture. Antibody concentrations were selected based on prior titration experiments demonstrating effective neutralization of exogenous IL‐2 (Supplementary Figure 2). Cells were harvested daily and analyzed for division. Additional aliquots were stained for activation markers (CD69, CD62L, CD127) and analyzed using flow cytometry (Supplementary table 2).

### Treg quantification

Thawed PBMCs were filtered through a 70 μm cell strainer, resuspended at 2 × 10^7^ cells/mL and Fc blocked (BD Biosciences). Cells were stained with a Treg antibody cocktail (Supplementary table 3) for 30 min on ice, washed and analyzed using a BD Fortessa X20 cytometer. Tregs were defined as CD3^+^CD4^+^CD8^−^CD25^+^CD127^low/^^−^ within the live lymphocyte gate.

### IL‐2 secretion and CD25 expression

Naïve CD4^+^ and CD8^+^ T cells were stimulated with anti‐CD3/CD28 Dynabeads either without IL‐2 (supernatant collected at 42 h) or with 100 U/mL IL‐2 for 42 h, followed by bead/supernatant removal and reculture in media alone for an additional 24 h (total ~70 h including wash and handling steps) and supernatant collected. Remaining cells from both conditions were stained with anti‐human CD25‐BV421 (Biolegend) and gMFI was measured by flow.

IL‐2 concentrations were quantified using V‐plex Human IL‐2 kits (Meso Scale Diagnostics) and read on a MESO QuickPlex SQ 120 plate reader (Discovery Workbench 4.0 software). The lower limit of detection (LLOD) was ~0.1 pg/mL per plate.

### Flow cytometry

Proliferation (CTV) kinetics were analyzed on a BD Canto II cytometer (Becton Dickinson). Calibration particles (CaliBRITE rainbow beads, BD Biosciences; 1 × 10^4^ beads/well) enabled absolute quantification. Dead cells were excluded using propidium iodide (0.2 μg/mL, Sigma‐Aldrich). For T‐cell activation markers, IL‐2R**α (**CD25) expression and Treg quantification, samples were analyzed on a BD LSRFortessa X20 cytometer. Hydroxystilbamidine methanesulfonate (1 μg/mL; Invitrogen, Thermo Fisher Scientific) was added immediately prior to acquisition for dead cell discrimination. All flow cytometry data were analyzed using FlowJo software (Treestar, version 10.10.0, Mac OS X) including gating strategies, population statistics and gMFI calculations.

### Cell counting and precursor cohort analysis

Absolute cell counts were calculated by scaling acquired events to calibration bead recovery:

$$\text{Total cell number}=\text{cells collected}\times \frac{1\times 10^{4}\;\text{total particles in well}}{\text{particles collected}}$$

Cell survival and division were quantified using the previously established precursor cohort method.^27^ The precursor cohort or initial pre‐stimulated undivided cells that contributed to each CTV peak were calculated as follows:

$$D_{i}=\frac{\;\text{number of cells counted in}\;i^{\mathrm{th}}\;\mathrm{CTV}\;\text{peak}}{2^{i}}$$

where i is the division generation number.

The total Cohort number and mean division number (MDN) were calculated as:

$$\text{Total Cohort Number}=D_{0}+D_{1}+D_{2}+\cdots +D_{i}$$

$$\mathrm{MDN}=\frac{\left(0\times D_{0}\right)+\left(1\times D_{1}\right)+\left(2\times D_{2}\right)+\cdots +\left(i\times D_{i}\right)}{\text{Total Cohort Number}}$$

Total cell numbers and total cohort numbers were expressed as fold‐change relative to initial cell seeding conditions; day 0 for 42‐h values, or day 2 for time points after stimulus removal. The area under the curve (AUC) was calculated using the normalized total cell number (relative to day 2) over the course of the experiment (day 2 to day 6). AUC was estimated using the standard trapezoidal rule for non‐uniformly spaced data points, $\left\{\left(t_{0},c_{0}\right),\left(t_{1},c_{1}\right),\ldots ,\left(t_{n},c_{n}\right)\right\}$, where $t_{j}$ and $c_{j}$ are jth harvested time point and normalized total cell number, respectively, such that $\mathrm{AUC}=\sum_{j=0}^{n}\left(\frac{c_{j}+c_{j+1}}{2}\right)\Delta t_{j}$ with $\Delta t_{j}=t_{i+1}-t_{i}$.

### Cyton2 modeling

T cell population dynamics were modeled using the Cyton2 framework and a standard fitting strategy to accurately capture the underlying cellular division timers.^16^ The model incorporates three lognormally distributed random variables (RVs): first division $T_{\mathrm{div}}^{0}\sim \mathrm{LN}\left(m_{\mathrm{div}}^{0},s_{\mathrm{div}}^{0}\right)$, time to death $T_{\mathrm{die}}\sim \mathrm{LN}\left(m_{\mathrm{die}},s_{\mathrm{die}}\right)$ and time to division destiny $T_{\mathrm{dd}}\sim \mathrm{LN}\left(m_{\mathrm{dd}},s_{\mathrm{dd}}\right)$ where $\left(m,s\right)$ denote median and log‐variance, respectively, of the lognormal distribution. A constant $b\in {\mathbb{R}}_{>0}$ represents the average time between subsequent divisions. The model was fitted to observed cell counts, denoted $\left\{n_{i,r}\left(t\right)\right\}$, where: $i\in \left\{0,1,\ldots ,I\right\}$ represents generation, $r\in \left\{0,1,\ldots R\right\}$ is a replicate index, $t\in T=\left\{t_{j}\in {\mathbb{R}}_{\geq 0}:j=0,1,\ldots ,J\right\}$ is discrete time points, and $I,R,J\in {\mathbb{Z}}^{0+}$ are the maximum observed generation, total number of replicates and final time point, respectively. The model contains seven parameters: $\theta =\left(m_{\mathrm{div}}^{0},s_{\mathrm{div}}^{0},m_{\mathrm{die}},s_{\mathrm{die}},m_{\mathrm{dd}},s_{\mathrm{dd}},b\right)$ for each set of measurements. We define the following cost function to minimize the residual sum‐of‐squares (RSS),

$$C\left(\theta \right)=\sum_{t\in T}\sum_{i=0}^{I}\sum_{r=0}^{R}{\left(n_{i,r}\left(t\right)-y\left(t;\theta \right)\right)}^{2},$$

where $y\left(t;\theta \right)$ is the model prediction. The minimization and best‐fit parameters were achieved by utilizing the least‐squares method and Levenberg–Marquardt Optimization algorithm^47^ such that

$$\left\{\theta^{*}\right\}\in \underset{\theta }{\arg\;\min}C\left(\theta \right),$$

$$\text{subjected to}\;m_{\mathrm{div}}^{0},m_{\mathrm{die}},m_{\mathrm{dd}}\in \left(0,500\right];s_{\mathrm{div}}^{0},s_{\mathrm{die}},s_{\mathrm{dd}}\in \left(0,2\right];b\in \left[0,50\right].$$

The best‐fit parameters were selected based on the minimum residual sum of squares (RSS), and model robustness was assessed across multiple initialization conditions. This process requires a set of parameter guesses to initiate the algorithm. Consequently, 100 uniform random sets of initial guesses were assigned for each parameter component within the identified ranges. Then, the best‐fit parameters were determined based on the corresponding lowest RSS value. Bootstrap resampling (*n* = 1000) was used to estimate 95% confidence intervals for the fitted parameters and generate confidence bands for model‐predicted cell numbers.^48^ Specifically, we constructed 1000 artificial datasets by resampling with replacement from the original dataset at each time point, allowing us to obtain an additional 1000 estimates to compute the errors.

### Statistical analysis

Due to cryopreserved PBMC use across multiple experiments, not all donors contributed data to each assay. CD4^+^ T cells were analyzed across both active and treated CeD cohorts, whereas CD8^+^ analyses were limited by sample availability. Statistical analyses were performed using GraphPad Prism (v9.5.0) unless otherwise stated. Welch's *t*‐test was applied for parametric comparisons. Linear and non‐linear curve‐fitting were applied where appropriate. Correlation analyses used Spearman's rank method. Statistical significance was defined as p<0.05. Where indicated, analyses were repeated in female‐only subsets to assess potential sex effects on T‐cell responses. For Cyton2 model parameters, a custom non‐parametric permutation test was implemented using Python v3.11.5 to evaluate differences between healthy and CeD donor estimates. Each parameter was assessed independently with null and alternative hypotheses defined as: $H_{0}:\left|\mu_{\mathrm{HD}}-\mu_{\mathrm{CD}}\right|=0;\text{and},H_{1}:\left|\mu_{\mathrm{HD}}-\mu_{\mathrm{CD}}\right|\neq 0,$ where $\mu_{\mathrm{HD}}$ and $\mu_{\mathrm{CD}}$ are the mean parameter estimates for healthy and CeD donor samples, respectively. Two‐sided *P‐*values were calculated using Monte‐Carlo simulations with $1\;\times \;10^{7}$ random permutations.

## CONFLICT OF INTEREST

VLB receives research funding from CSL and Immunosis and consults for Immunosis. JT‐D has privately or via his institute been a consultant or advisory board member for Anatara, Anokion, Barinthus Biotherapeutics, Chugai Pharmaceuticals, Dr. Falk, Equillium, EVOQ Therapeutics, IM Therapeutics, Janssen, Kallyope, Mozart Therapeutics, Takeda, TEVA and Topas and has received research funding from Barinthus Biotherapeutics, Chugai Pharmaceuticals, Codexis, DBV Technologies, EVOQ Therapeutics, Immunic, Kallyope, Novoviah Pharmaceuticals, Topas and Tillotts Pharmaceuticals. He is an inventor on patents relating to the use of gluten peptides in coeliac disease diagnosis and treatment. MYH is a consultant for Takeda. The remaining authors have declared that no conflict of interest exists.

## ETHICS APPROVALS

All study protocols were approved by the Human Research Ethics Committees of Melbourne Health HREC/16/MH261, 2009.162 and 2020.162 and WEHI (10/02). All participants provided written, informed consent in accordance with the Declaration of Helsinki and its later revisions.

## AUTHOR CONTRIBUTIONS

**Anthony J Farchione:** Methodology; investigation; formal analysis; visualization; writing – original draft. **David Vremec:** Methodology; investigation. **Lee M Henneken:** Investigation. **Julika Neumann:** Methodology; investigation. **Lauren J Howson:** Investigation. **HoChan Cheon:** Methodology; investigation; formal analysis; visualization; writing – original draft; software. **Maureen Forde:** Investigation. **Philip D Hodgkin:** Conceptualization; methodology; formal analysis; writing – original draft; supervision; funding acquisition; resources; data curation. **Gwenny M Verstappen:** Methodology; investigation; writing – review and editing. **Melinda Y Hardy:** Methodology; investigation; writing – review and editing. **Mai B Margetts:** Methodology; investigation. **Susanne Heinzel:** Conceptualization; methodology; formal analysis; writing – original draft; supervision; data curation. **Vanessa L Bryant:** Conceptualization; methodology; formal analysis; writing – original draft; supervision; resources; funding acquisition; software; data curation. **Jason A Tye‐Din:** Writing – original draft; resources; funding acquisition.

## FUNDING

This work was supported by the Snow Medical Foundation through the Snow Centre for Immune Health and NHMRC Grant funding (APP1127198 and APP1164800). VLB was supported by Sir Clive McPherson Family Fellowship, DW Keir Fellowship. JT‐D was supported by an NHMRC Investigator Grant (APP1176553). PDH was supported by NHMRC Investigator Grant (APP1176588). This work was made possible through the Victorian State Government Operational Support Program and the Australian Government NHMRC IRIISS.

## Supporting information


Supplementary figure 1.
**Supplementary figure 2**.
**Supplementary figure 3**.
**Supplementary figure 4**.
**Supplementary figure 5**.
**Supplementary figure 6**.
**Supplementary figure 7**.
**Supplementary figure 8**.
**Supplementary figure 9**.
**Supplementary figure 10**.
**Supplementary table 1**.
**Supplementary table 2**.
**Supplementary table 3**.

Data S2.

## Data Availability

Supporting data values are provided in the Supporting Data Values spreadsheet. Precursor cohort data, including individual donor samples, cell types (CD4^+^ or CD8^+^) and conditions (media alone, anti‐IL‐2/IL‐2R**α** and anti‐IL‐2/IL‐2R**α**+rhuIL‐2), used to generate proliferation analyses and Cyton2 modeling outputs, are available at: https://github.com/hodgkinlab/CeDmomentum2025. This repository also includes Cyton2 model parameter estimates (medians and standard errors) for each cellular timer and figures of the fitted lognormal distributions for all donor responses.
